# The association between a free medicine program and functioning in people with schizophrenia: a cross-sectional study in Liuyang, China

**DOI:** 10.7717/peerj.8929

**Published:** 2020-04-24

**Authors:** Wenjie Gong, Chao Zhang, Dong (Roman) Xu, Shuiyuan Xiao, Yu Yu, Eric D. Caine

**Affiliations:** 1 Xiangya School of Public Health, Central South University, Changsha, Hunan Province, China; 2 Sun Yat-sen University Global Health Institute (SGHI), School of Public Health & Institute of State Governance, Sun Yat-sen University, Guangzhou, Guangdong Province, China; 3 Xiangya Hospital, Central South University, Changsha, Hunan Province, China; 4 Department of Psychiatry, University of Rochester, Rochester, NY, USA

**Keywords:** Free medication program, 686 Program, Medication adherence, Community-based mental health

## Abstract

**Background:**

Persons with schizophrenia frequently discontinue or avoid medications. Under a national community-based mental health program many places in China have started to provide free medications to people with severe mental health disorders in their communities. In the free medication program (FMP) in Liuyang, China, peripatetic psychiatrists prescribed and dispensed antipsychotic medications free of charge at regular intervals and places convenient for all patients through the primary health care system since 2006. Our study aims to test whether adherence to this FMP improves the functioning of people with schizophrenia.

**Methods:**

The research was conducted in Liuyang, a rural county in central China. Data were obtained from three sources: an ad-hoc survey and the program’s management system in 2013 and in-home interviews in 2014 in Liuyang. We conducted a cross-sectional propensity-score based analysis of the “dose” effect of FMP participation on their level of functioning, using medication refill adherence as a proxy for the participatory intensity in the program.

**Results:**

Only 50.9% of 2,332 participants came for all refills in 2012. Higher refill adherence was associated with slightly worse functional outcomes. The main reasons for refill non-adherence were “unwilling to take medication or only took medication when unwell” (23.6%), “forgot or missed the appointment” (20.6%) and “hospitalized” (20.1%).

**Conclusions:**

The FMP program in Liuyang seemed to have successfully removed barriers in cost and access. However, better refill adherence was not associated with better functional outcomes in this study, which might have been the result of reverse causation. To improve the effectiveness of the FMP, patient-centered measures should be considered.

## Introduction

With a worldwide prevalence of 1%, schizophrenia challenges families, communities and health systems and exemplifies the complexities posed by complex non-communicable diseases (Mueser & McGurk, 2004). Maintaining patient functioning and reducing symptoms often depends upon the continuous use of antipsychotic medications (Leucht et al., 2013). However, persons suffering from schizophrenia frequently discontinue or avoid needed medications (Knapp et al., 2004; Razali & Yahya, 1995). This problem may reflect at least four barriers—prohibitive cost, difficult access, individual acceptance and side effects that diminish willingness to continue treatments (Velligan et al., 2010). Providing freely available medicines in relatively convenient local settings may offer effective solutions to overcome common challenges that confront many people living in resource-limited settings. Whether improving the accessibility of medication would increase adherence and improve health outcomes is an interesting question. In the Clinical Antipsychotic Trial of Intervention Effectiveness, in addition to their main finding on the difference of effectiveness between older and newer generations of antipsychotic drugs, they found that 74% of patients overall had stopped taking the trial medication before 18 months (Lieberman & Stroup, 2011; Lieberman et al., 2005). Surprisingly it may seem but this observation was actually similar to other programs that provided free antipsychotic medication (Chen, McCombs & Park, 2008; Kilzieh et al., 2008; Moisan & Grégoire, 2010; Rothbard, Kuno & Foley, 2003). In China, the implementation of “the National Continuing Management and Intervention Program for Psychoses” (also known as the “686 Program”) provided an opportunity to revisit the question of whether improving access would result in increased compliance and outcome. The “686 Program” covers six severe mental health disorders including schizophrenia. It began in 2004 and later evolved into the national essential public health service (Song et al., 2011). Under this policy, many sites in China have started to provide free medications to people with severe mental health disorders in their communities, often as the result of the additional local investment into the program.

Liuyang is a rural county of Hunan province in China and has been one of the first 30 counties participants in the“686 Program”. Free Medication Program (FMP) has operated there since June 2006. Currently, the FMP support includes several streams of funds: “686 Program” from the central government supports program administration and training; regional funds are used to pay for medications and the Liuyang City civil affairs department pays for the travel expenses of medical teams that work through the county. The government of Liuyang has for long prioritized mental health work and has allocated substantial human, financial and organizational resources to this program. The experience in this area may well be useful for other places.

We surveyed all patients with schizophrenia registered in Liuyang FMP. The aim was to examine the reasons that FMP participants did not refill their medications and to explore relationships between FMP and patient functioning. We hypothesized that a “dose-response” may exist in that the participants with higher program participatory intensity had better functional outcomes than those with lower program participatory intensity.

## Materials and Methods

### Settings

Eighty-nine percent of Liuyang County’s 1.4 million residents live in 28 rural townships. The net income per capita was approximately USD 2,500 in 2012. In 2006, Liuyang integrated mental health service funding from central, provincial and municipal governments to launch FMP (Song et al., 2011). The Liuyang health department provided overall program supervision while delegating management and operation to the public-funded Liuyang Mental Health Hospital. A psychiatrist served as the full-time program director, supported by two other psychiatrists and several nurses working part-time (Gong et al., 2014). All “psychiatrists” were internists who converted their roles through on-the-job training. The psychiatrists together with several nurses traveled every 2 months to each township health center (THC) to provide patient consultation and medication. The township mental health administrators (MHAs), supervised by both the psychiatrists and local THCs, coordinated the work of “village doctors” to provide regular services, which included yearly physical exams, assessment of risk levels, at least home visits a year, health education and urgent care. In rural Liuyang county, MHAs and village doctors typically have received 2–3 years of medical or public health training after middle school or high school.

### Data

Three sources of data were used. First, we retrieved the social-demographic information and collected information regarding their illnesses and treatments in 2012 including the medicine refill record, date of onset of the condition, date of diagnosis, relationship with the informant, hospitalization and outpatient attendance in 2011, medication adherence, duration of participation in the FMP, the frequencies of relapse requiring hospitalization from the FMP management system. The refill times were used as a proxy for the level of intensity of participating in FMP: out of the six required refills in 2011, attending at least five was regarded as “refill-adherent”. Second, the MHAs administered a brief survey to the key informants (typically family members) of all FMP enrollees regarding their adherence and functional information in early 2013. The survey covered functional evaluation in three domains: employment, daily living and interpersonal relationships. The functional disability in each domain was rated on a 5-point scale ((1) Broadly similar to healthy individuals. (2) Slightly less functional than healthy individuals. (3) Half as functional as healthy individuals. (4) Less than half as functional as healthy individuals. (5) Basically not functional at all). The sum of the score of each domain (the least disabled score of 3 to the most disabled of 15) was used as an indicator of the overall functional capacity. Adherence to medication in the past 6 months and past 1 month was assessed as follows: (1) Complete adherence daily. (2) Adherence on most days. (3) Adherence half of the time approximately. (4) Less than half of the time. (5) Take almost no medication. Finally, we conducted household interviews in four randomly selected townships to assess medication refill history from 2011 to 2013 and reasons for missing refills in July 2013. We collected 199 responses out of 226 FMP participants. Informed consent for participating in the surveys was obtained from all participants.

The four townships were randomly selected with the simple random sampling method. Once the townships were randomly selected, we found a total of 315 program enrollees. From 2011 to 2013, only 89 out of those 315 patients came for each of the scheduled refills for their medications. We made a home visit to each of the 226 people who missed some of their refills and succeeded in obtaining the reasons for their missing the refills from 199 people—27 of the 226 were not at home or declined our interview. All the instruments used in the study were self-development and implemented by our trained master degree public health students. Although those instruments were not validated, we consulted widely with various stakeholders and experts and piloted them in several sites before official fielding. The final form of the instruments was revised in accordance with the feedback we have received. The household interviews were conducted by the first author of this article and one trained master degree public health student.

### Analysis

We first performed descriptive analyses. Then we compared the functional levels of FMP enrollees between the refill-adherent and non-adherent groups, using propensity score analysis: we first calculate a “propensity score” for each participant; and then conduct a regression analysis to examine the relationship between functioning and refill-adherence, with those propensity scores included as covariates to control for known confounders (Xu et al., 2018). The propensity score is the probability of being assigned into an “exposure” conditional on observable covariates. Specifically to our study, the propensity score is the probability of being “assigned” to the refill-adherent group, the group with higher participatory intensity of the “686” enrollees (exposure of interest), conditional on a wide range of variables that may affect patient function (the outcome) or that may affect both the function and the “assignment” (the confounding factors). The calculation of the propensity score used 21 variables, including: participants’ sex, job, age, ethnicity, marital status, education, number of family members, onset date of schizophrenia, diagnosis date, person who was primary caregiver, hospitalization history due to the illness, outpatient attendance, hospitalization times in 2011, frequency of medication refills in 2011, medication adherence in the past 6 months, days of taking medicine per month, person who managed medication for patient, person who supervised medication-taking, family income in 2011, health insurance status, and medical expenses in 2011.

After patients were assigned a propensity score, their functional outcomes were compared between the two groups of patients of different FMP participatory intensity but otherwise in the same quintile of propensity scores. Though unobserved characteristics were not captured by the propensity score, including the propensity scores in the analysis allowed us to make a fairer comparison of functions between the two sets to examine the hypothesis that with other things being equal, participants with higher program participatory intensity had better functional outcomes than those with lower program participatory intensity. STATA 12 was used for the analysis.

## Results

### Characteristics of the study participants

The FMP covered 2,332 participants in 2012: the mean age was 42; 1,364 were women (of whom 78.0% were married) and 968 men (35.5% married). The level of education was generally low, with 54% of participants only having primary school education or below. 2,052 (87.9%) were farmers and 2,187 (93.8%) were receiving family care. Functional disability scores ranged from 3 to 15 (Table 1), with an overall mean of 8.17 ± 4.16 (*n* = 2,126), female mean of 8.12 ± 4.16 (*n* = 1,249) and male mean of 8.25 ± 4.16 (*n* = 877).

**Table 1:** Functional Loss (*n* = 2171).

	#	%	Cumulative (%)
**WORK**			
Almost no loss	553	25.57	25.57
Some loss	542	25.06	50.62
Half-lost	300	13.87	64.49
More than half loss	305	14.1	78.59
Total loss	463	21.41	100
**DAILY LIVING**			
Almost no loss	621	28.6	28.6
Some loss	569	26.21	54.81
Half-lost	344	15.85	70.66
More than half loss	362	16.67	87.33
Total loss	275	12.67	100
**SOCIAL INTERACTION**			
Almost no loss	532	24.49	24.49
Some loss	574	26.43	50.92
Half-lost	302	13.9	64.83
More than half loss	342	15.75	80.57
Total loss	422	19.43	100

### Association of program participation and functioning

The group with higher refill-medication adherence had a small but statistically significant degree of poorer functioning as compared to its less adherent peer group in 2011 (*p* = 0.015), after using propensity score to control for a range of potential confounders including socioeconomic and demographic variables, severity of disease and family support and engagement in care, the functioning score in the adherent group is 8.6 (95% CI [8.16–9.04]) (*p* < 0.001) and in the non-adherent group it is 7.7 (95% CI [7.26–8.22]) (*p* < 0.001).

### The reason for missing medication refills

Only 50.9% of the FMP participants came for each of the six free medication refills and 8.9% of participants did not present at any refills in 2011 (Table 2). Our survey of 199 FMP participants who had missed at least one refill suggested that patient preference and forgetfulness were two top reasons for not refilling their prescription (Table 3). Almost 14% chose to purchase their own drugs despite the availability of free medications.

**Table 2:** Medication refills condition of schizophrenic patients in Liuyang City in 2011 (*n* = 1,874).

Frequency of medication refills (2011)	*N*	Ratio (%)	Cumulative ratio (%)
0	168	8.96	8.96
1	85	4.54	13.5
2	137	7.31	20.81
3	139	7.42	28.23
4	230	12.27	40.5
5	161	8.59	49.09
6	954	50.91	100

**Table 3:** Reasons of missed refills among FMP participants in 4 Towns of Liuyang (*n* = 199).

Reasons	Numbers of respondents (*n*)	Percentage*
Do not want to/only take medicine when feeling ill	47	23.6
Forgot the refill appointments	41	20.6
Hospitalized	40	20.1
Partial adherence to dosages leading to left-over pills from the last refill	39	19.6
Self-purchase of medications	28	14.1
Feel well/no illness	20	10.1
Medication leading to side effects	16	8.0
Living outside of the towns (as migrant workers or doing business)	11	5.5
Medications have no effect	9	4.5
Medication-taking not supervised	7	3.5
Wander away	3	1.5
Others (pregnancy etc)	5	2.5

**Note:**

* The sum of percentage exceeds 100% due to respondents could choose several reasons.

## Discussion

The Public Health Service Package that includes the original “686 Program”, now covers entire China and thus made it impossible to evaluate the effect of “686 Program” with rigorous designs such as randomized controlled studies (Ma et al., 2011; Zhu et al., 2014). We believe that this study provides some unique insights into the effectiveness of this vast national program. We found only 50.91% of participants came for each refill. The group with higher refill adherence had a small but statistically-significant poorer functioning as compared to the less adherent group. Better refill adherence was not associated with better functional outcomes in our sample. The main reasons for refill non-adherence were “unwilling to take medication or only took medication when unwell” (23.6%), “forgot or missed the appointment” (20.6%) and “hospitalized” (20.1%).

**Figure 1:**
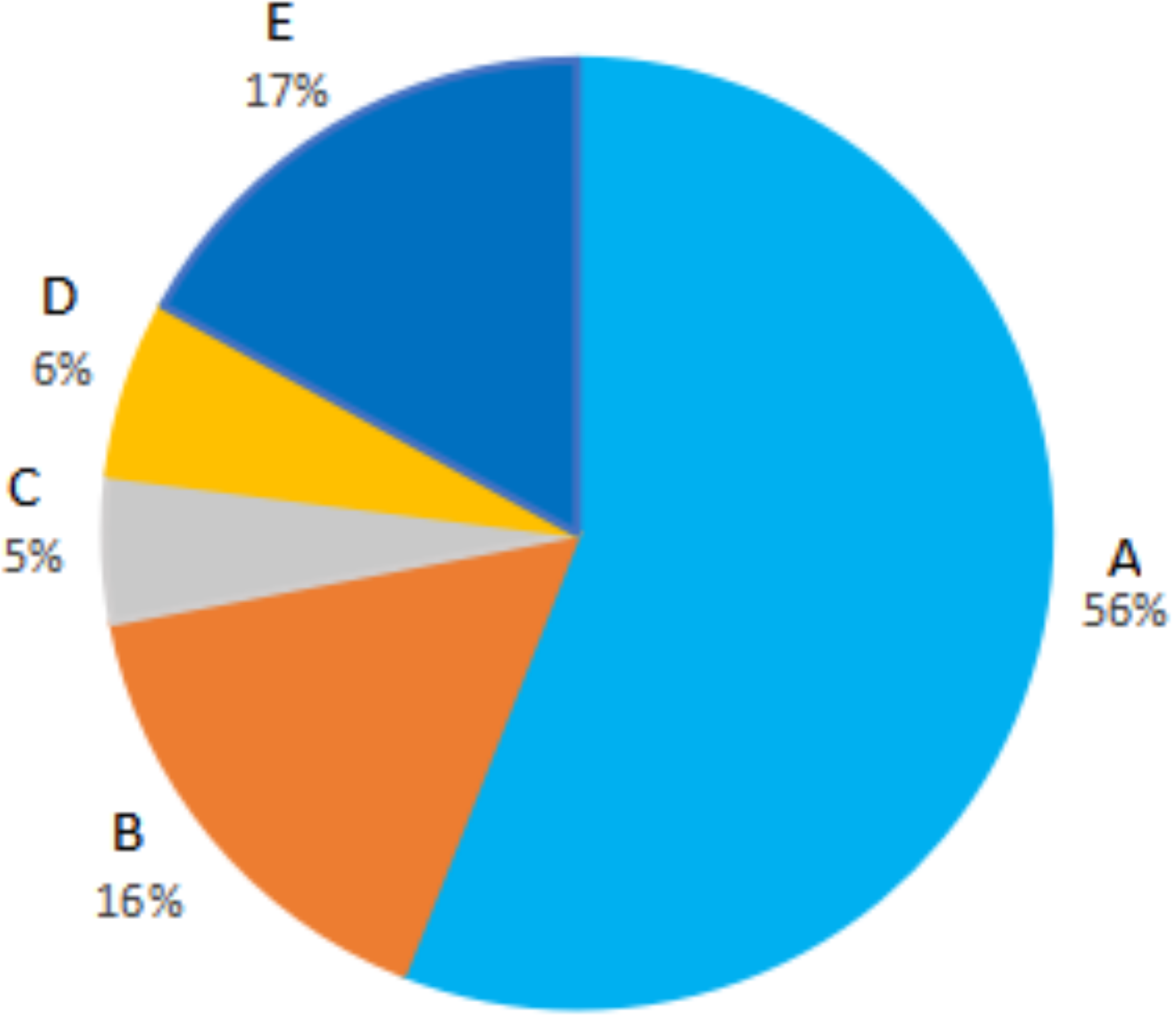
Self-report medication adherence for the recent 6 months. (A) Taking medications daily. (B) Taking medications most of the time. (C) Taking medications half of the time. (D) Taking medications less than half the time. (E) Taking almost no medications.

Social and demographic data of our FMP participants are similar to the national average of “686 Program” participants. Using the MHAs’ data from 2013, FMP participants in Liuyang reported substantially greater adherence during the prior 6 months as compared to the national average (56% versus 24% taking medication daily) and better than many other places, including Beijing (46%) (Li et al., 2012; Wang et al., 2016). The self-reported adherence (Fig. 1) was similar to that obtained through the home-based pill count in 2016 (Xu et al., 2018). The rate of hospitalization in 2012 in Liuyang was 13%, which compared favorably to many other “686” localities (Bai et al., 2011; Wen et al., 2011). The program in Liuyang seemed to have successfully removed barriers in cost and access that may have resulted in higher adherence and reduced hospitalization.

Some practices in Liuyang may explain the relative strengths of the Liuyang program. First of all, the antipsychotics were offered for free to all program participants in Liuyang while only the people meeting certain conditions such as the poverty line received free medication in most other places. Second, Liuyang has been one of the earliest participants in the 686 Program and is likely to have accumulated more program experiences than other places. Lastly, the government of Liuyang has for long prioritized mental health work and has allocated more human, financial and organizational resources to the mental health program.

We found that greater refill-adherence was not associated with better functioning in our sample. There are several plausible explanations: firstly, a higher refill adherence in those whose condition was more severe was not as unexpected as patients or their families may have been more keen to seek better control with medication. In other words, our observation was the result of reverse causation. As we do not collect information on baseline followed by follow up, this potential explanation cannot be confirmed or refuted. Also, getting a refill did not equate to taking the medication. While the FMP addressed barriers associated with cost and access, it did not overcome those “patient-centered” matters such as side effects that matter to the patient, the stigma associated with taking the medication and cognitive deficiency that leads to forgetfulness of taking medications.

In the survey we conducted in the four counties, we found that the main reasons for refill non-adherence were “unwilling to take medication or only took medication when unwell” (23.6%), “forgot or missed the appointment” (20.6%) and “hospitalized” (20.1%). To improve the effectiveness of the FMP, efforts to modify the negative attitudes among some patients towards antipsychotic medications and to explain the benefits of medications would be useful. There is also good evidence that reminders may improve compliance (Wen et al., 2011). This may be particularly worth exploiting as 80% of people with schizophrenia in China believed that medication helps but over half of them felt that they often forgot to take them (Dixon et al., 2009; Lincoln, Wilhelm & Nestoriuc, 2007; Pekkala & Merinder, 2002; Van Dulmen et al., 2007; Zygmunt et al., 2002). In this aspect, the wide use of smartphones and mobile health may help. Indeed, our trial has shown that mobile texting significantly improved patient adherence to antipsychotics in Liuyang (Xu et al., 2019).

Despite its imperfection, this relatively low-cost community-based free medicine solution in Liuyang may provide an example in mental health for other low and middle-income countries. The program with free medication as its core has several features that may have significant implications for the countries with limited resources. First, the model of free medications provided by touring psychologists, nurses and the medication van on fixed dates and fixed locations near where people live may reduce access to care and medication. Second, the program is kept at low cost, with mostly inexpensive medications and the use of existing health system structures. Third, it is collaborative care among psychiatrists, MHAs, village doctors and the family members and patients with cascading tasks of care appropriate for each level. Finally, the way of program management and financing enables multi-agency (health, civil affairs, neighborhood, family and police, etc.) approaches to deal with severe mental disorders. Elements of the program can potentially be implemented in many other low and middle-income countries where human resources for mental health is scarce and medications are not easily accessible.

Our study has several limitations. First, we use “dose-response” as a proxy for program effect, but the linkage between this proxy and the actual effect may not be definitively established. Second, even though we have used a propensity score to try to control for as many as possible potential and available confounders, we cannot control for unobservable and unavailable confounders. Third, as we surveyed a large sample, we used a simple question to assess patient function as complicated functional assessment was infeasible. Finally, due to limitations in our data, it is hard to examine the true association between the free medicine program and functioning in patients with schizophrenia in Liuyang.

## Conclusions

Free antipsychotic medications, routinely and conveniently delivered through the public health service to persons suffering schizophrenia, removed barriers related to cost and access. Medication refill was higher and hospitalization was lower compared to many other regions in China. However, better refill adherence alone was not associated with better functional outcomes in this study. Comprehensive approaches may be needed to address patient preferences, unwanted side effects, suboptimal treatments and other rehabilitative needs (Xu et al., 2016, 2019). Local support and implementation are vital to complement the national program.

## Supplemental Information

10.7717/peerj.8929/supp-1 Supplemental Information 1 686 Program Data.

10.7717/peerj.8929/supp-2 Supplemental Information 2 Information investigation form of Patients with Severe Mental Diseases in Liuyang City.

## Additional Information and Declarations

### Data Availability

The following information was supplied regarding data availability: The raw data are available as a Supplemental File.
