# Supplementary material for: The association between a free medicine program and functioning in people with schizophrenia: a cross-sectional study in Liuyang, China
Source: PeerJ. 2020 Apr 24;8:e8929. doi: 10.7717/peerj.8929 (PMC7185026; doi:10.7717/peerj.8929)
Supplement: Supplemental Information 2 [file peerj-08-8929-s002.docx]

**Information investigation form of Patients with**

**Severe Mental Diseases in Liuyang City**

*Severe Mental Diseases including the existing registered six categories of mental disorders : schizophrenia, paranoid mental disorders, epilepsy-induced mental disorders, emotional disorders, schizo-affective, retardation.*

1. **Patient General information**
2. **Full Name***(Name corresponds to ID card)*：
3. **Gender：**□male / □female
4. **Chronological Age:：**

*(Note:Chronological age equals 2012 minus the year of birth.)*

1. **Diagnosis/Diagnoses：**

*(Note:Please show patient’s medical record books to the investigators,they need to check the diagnosis/diagnoses .)*

**a.Year of Onset** *(when family members notices the abnormality)***：**

**b.Year of Diagnosis/Diagnoses***（when the hospital has given a definite diagnosis/diagnoses）.***：**

**(3)Ethnicity:** Han/Minority*(Please specify what kind of minority it is.)***：**

**(4)Detailed Address***( The address that we can use to find patient's residence.)***：**group（resident committee） ，village（street） ，county（township）

1. **Contact Name：** ，**Contact phone**：

*(Note: About “contact phone”.It is better to fill in the cell phone number. We will check the accuracy of the information by calling. If you have a telephone number, please fill in the telephone number next to it.)*

**Education Background：**

①elementary school and below

②junior high school

③[high school](http://dict.cnki.net/javascript:showjdsw('showjd_0','j_0'))/vocational senior school/technical secondary school

④junior college

⑤Bachelor degree and above

**(7)Marital Status：**

①[unmarried](http://dict.cnki.net/javascript:showjdsw('showjd_0','j_0'))*(no marriage/no cohabitation/single )*

②married*(first married /remarried）*

③divorced

④widowed

⑤unmarried but cohabit*(Haven’t been married at a registry, but live together.)*

**(8)If the patient has a spouse now, fill in the following information:**

*(Note:If the patient has a cohabiting spouse, you also need to fill in the following three questions.)*

1. Whether the spouse is first married to the patient: □Yes/□No
2. Spouse's body condition: □ health / □disability
3. Registered location of spouse's pre-marital residence:

①Liuyang City; ②Other places in Hunan Province; ③Not in Hunan Province.

1. **Who is the main caregiver of the patient?**
2. **Occupational type：**①agricultural related work ②agricultural unrelated work ③student
3. **Is there any interruption of study or work due to illness at present?**□**Yes/**□**No, if the answer is "Yes", from which year?**
4. **Patient's condition and treatment**

（1）**Hospitalization information of patients：**

①Never been hospitalized ②Have been hospitalized, totally times， including:

times,in the year of

times,in the year of

times,in the year of

times,in the year of

times,in the year of

times,in the year of

*(Note:Try to recall the number of hospitalizations as much as possible. If it's too early to recall, at least from 2005 to now.)*

（2）**Have you ever committed suicide?**①Never committed suicide②Yes,the patient have committed suicide for ________ times，including：

times,in the year of

times,in the year of

times,in the year of

times,in the year of

times,in the year of

times,in the year of

（3）**Patient Runaway Information：**①never runaway ②have the experience of runaway，totally times，including：

times,in the year of

times,in the year of

times,in the year of

times,in the year of

times,in the year of

times,in the year of

*（Note：If the patient leaves home alone for more than one night without the knowledge of family members,no matter near or far,it can be counted as a runaway.）*

1. **Have the patient joined the Government's Free Medicine Assistance Program?**

□Yes/□No

*（Note:If you choose "No", you should jump directly to question 5. If you choose "Yes", you should first answer the following questions:① and ②, and then answer question 5 and other questions.）*

a.The year of joining the project： .

b. In 2011, how many times have you received the government's free medicine?

times.

*（Note：The medicines have been distributed for six times from January to November 2011. How many times of them have you received?）*

**（5）Have the patient ever visited psychiatric clinics in 2011：**①No ②Yes，the number of times is ___________*(excluding receiving medicine at drug-distribution points).*

**（6）Have the patient ever been hospitalized in 2011：**

①No ②Yes, ________ times.

**（7）Have the patient ever losen his/her temper and beatten other people in 2011：**

①No ②Yes, ________ times.

1. **Have the patient ever losne his/her temper and thrown things in 2011：**

①No ②Yes, ________ times.

（9）**In 2011, the amount for the disease（including outpatient, hospitalization, medicines, transportation, etc.)at own expenses was approximately**:

yuan.

*(Note:“The disease” should be consistent with the previous "diagnosis/diagnoses" in the general information.)*

1. **Taking medicine in the last six months：**

①takes medicine every day；②most of the time adhere to medication；

③about half of the time adhere to medication；④less than half of the time adhere to medication；⑤Generally doesn’t take medicine.

1. **The number of days that patient have taken medince in the last month (30 days)** .
2. **Taking medicine management** *(including drug purchase, receipt, storage, etc.).***:**

①mainly patient self-management; ② mainly other people in the family *(please explain the relationship between the person who manages the medcine and the patient)* .

1. **Taking medicine supervision***(including daily urge patient to take medicine, calculation of dosage, etc.)***:** Yes/No, if there is someone urges the patient to take the medicine,his/her relationship with the patient is  *.*

*(Note:if you choose "no", you don't need to fill in, if the patient urges himself, fill in "myself".)*

1. **At present, the main therapeutic medicine is** .

*(Note:Please take out the medicine that patient is taking recently,we need to check it.)*

1. **The main method of medical payment：**

①at own expenses ②New Rural Cooperative Medical Insurance System ③Residents Medical Insurance ④Urban and Rural Residents Medical Insurance

⑤Medical Insurance for Urban Workers ⑥Business Supplement Medical Insurance

1. **Please compare the general status of the patients with that of the residents in their villages, and asses***(self-assess or family member assess)***the working ability of the patients according to the results of the comparison：**

①generally the same as the ordinary people ②a little worse than the ordinary people

③almost half the ordinary people ④less than half of the ordinary people

⑤generally incapable of working

1. **Please compare the general status of the patients with that of the residents in their villages, and asses***(self-assess or family member assess)* **the daily activities of the patients according to the results of the comparison：**

①generally the same as the ordinary people ②a little worse than the ordinary people

③almost half the ordinary people ④less than half of the ordinary people

⑤generally incapable of daily living

1. **Please compare the general status of the patients with that of the residents in their villages, and asses***(self-assess or family member assess)* **the interpersonal communication ability of the patients according to the results of the comparison：**

①generally the same as the ordinary people ②a little worse than the ordinary people

③almost half the ordinary people ④less than half of the ordinary people

⑤generally incapable interpersonal communication

1. **Family information of patients**

(1)**How many family members?** .

(2)**Family Members Information：**

*（Note:In terms of occupational type, please fill in the following options: ①agricultural related work ②agricultural unrelated work ③student）*

| **Relationship with patients** | **Chronological Age** | **Education Background** | **Gender** | **Occupational type** | **Health status**  *（If the patient have mental disorders, please specify the diagnosis/diagnoses,and show patient’s medical record books to the investigator.）* |
| --- | --- | --- | --- | --- | --- |
|  |  |  |  |  |  |
|  |  |  |  |  |  |
|  |  |  |  |  |  |
|  |  |  |  |  |  |
|  |  |  |  |  |  |
|  |  |  |  |  |  |
|  |  |  |  |  |  |
|  |  |  |  |  |  |

1. **Family income (gross income) in 2011 was:** yuan.

*（Note：Please give a rough estimate.）*

(4)**The main income source of family members in 2011 are：***(Multiple selection)*

①planting crops ②working ③doing business ④livestock breeding ⑤Fishery ⑥unit wage ⑦government subsidy ⑧ family support ⑨others：

1. **Family total cost for Medical Care:** yuan.

*(Note:The direct costs of all illnesses for all family members are included.)*

**(6)If the patient has children, please answer the following questions:**

a.Does the patient live with his/her children?□Yes**/□**No

b.The main caregiver of children is

①Patient himself/herself

②Not the patient himself/herself,and the relationship between the patient and the caregiver is .

1. **Total cost of education for all the children in 2011 (tuition, stationery, books, etc.)：**  yuan.
